# *AtSK11* and *AtSK12* Mediate the Mild Osmotic Stress-Induced Root Growth Response in *Arabidopsis*

**DOI:** 10.3390/ijms21113991

**Published:** 2020-06-02

**Authors:** Long Dong, Zhixin Wang, Jing Liu, Xuelu Wang

**Affiliations:** 1 College of Life Science and Technology, Huazhong Agricultural University, Wuhan 430070, China; donglong063@126.com (L.D.); wxddzyx01@163.com (Z.W.); jingliu95@foxmail.com (J.L.); 2 State Key Laboratory of Crop Stress Adaptation and Improvement, School of Life Sciences, Henan University, Kaifeng 475000, China

**Keywords:** GSK3, water potential, osmotic stress, root growth, *Arabidopsis thaliana*

## Abstract

Although most osmotic stresses are harmful to plant growth and development, certain drought- or polyethylene glycol (PEG)-induced mild osmotic stresses promote plant root growth. The underlying regulatory mechanisms of this response remain elusive. Here, we report that the GLYCOGEN SYNTHASE KINASE 3 (GSK3) genes *ARABIDOPSIS THALIANA SHAGGY-RELATED KINASE 11* (*AtSK11*) *(AT5G26751*) and *AtSK12* (*AT3G05840*) are involved in the mild osmotic stress (−0.4 MPa) response in *Arabidopsis thaliana*. When grown on plant medium infused with different concentrations of PEG to mimic osmotic stress, both wild-type (WT) and *atsk11atsk12* plants showed stimulated root growth under mild osmotic stress (−0.4 MPa) but repressed root growth under relatively strong osmotic stress (−0.5, −0.6, −0.7 MPa) as compared to the mock condition (−0.25 MPa). The root growth stimulation of *atsk11atsk12* was more sensitive to −0.4 MPa treatment than was that of WT, indicating that *AtSK11* and *AtSK12* inhibit the mild stress-induced root growth response. RNA-seq analysis of WT and *atsk11atsk12* plants under three water potentials (−0.25 MPa, −0.4 MPa, −0.6 MPa) revealed 10 differentially expressed candidate genes mainly involved in cell wall homeostasis, which were regulated by *AtSK11* and *AtSK12* to regulate root growth in response to the mild stress condition (−0.4 MPa). Promoter motif and transcription factor binding analyses suggested that the basic helix-loop-helix (bHLH) transcription factor bHLH69/LJRHL1-LIKE 2 (LRL2) may directly regulate the expression of most −0.4 MPa-responsive genes. These findings indicate that mild osmotic stress (−0.4 MPa) promotes plant growth and that the GSK3 family kinase genes *AtSK11* and *AtSK12* play a negative role in the induction of root growth in response to mild osmotic stress.

## 1. Introduction

Plants live in constantly changing environments, which sometimes can be unfavorable for plant growth and development. In addition, climate changes and extreme water conditions are becoming more intense [1]. The sessile nature of plants requires that they evolve complex mechanisms to cope with various stress conditions such as drought-induced osmotic stress. Under severe osmotic stress, plants usually accelerate their life cycle to ensure reproduction before death, whereas under mild osmotic stress, such as moderate drought, plants usually adjust their growth to balance water uptake and water loss, and, more importantly, enhance root growth to promote water uptake [2,3,4]. 

Drought- or polyethylene glycol (PEG)-induced mild osmotic stress facilitates plant root growth. It is reported that drought stress during later stages of vegetative growth or early reproduction development increases root growth in soybean [5]. Similarly, in cotton, mild drought stress during the initial stage enhances root elongation [6]. A method was developed for producing plant medium to impose different osmotic stress conditions by introducing PEG into solidified medium through diffusion [7]. Notably, mild osmotic stress (near −0.2 MPa) stimulates primary root growth of *Arabidopsis* seedlings and, in light-grown seedlings, root elongation decreases under relatively strong osmotic stress conditions (water potentials below −0.5 MPa) [7]. In addition, *Arabidopsis* root growth increases slightly in the PEG-infused half-strength Murashige and Skoog (MS) medium (−0.75 MPa) compared to the mock condition (−0.3 MPa), whereas root growth is severely inhibited in salt-containing medium with the same water potential (−0.75 MPa) [8]. These findings indicate that the root growth response under PEG-mimicked osmotic stress is different from that under salt stress, which causes both ion stress and osmotic stress. However, to date, how moderate osmotic stress facilitates root growth remains unclear.

Osmotic stress signal-transduction networks have been well studied. In the cell membrane, REDUCED HYPEROSMOLALITY-INDUCED CALCIUM INCREASE 1 (OSCA1) forms hyperosmolality-gated calcium-permeable channels to induce increases in cellular [Ca ^2+^] and mediate signal transduction in response to osmotic stress [9]. In response to osmotic stresses, levels of phytohormone abscisic acid (ABA) rapidly increase in plants under osmotic stress [10]. The ABA binds to its receptors and to protein serine/threonine phosphatase 2Cs (PP2Cs), thereby facilitating the activation of SNF1-RELATED PROTEIN KINASE 2s (SnRK2s), which in turn activate the b-ZIP transcription factors ABA-responsive element (ABRE)-binding proteins/ABRE-binding factors (AREB/ABFs) to regulate stress-responsive gene expression [11]. Osmotic stress also activates lipid signaling and induces the expression of genes such as *PHOSPHATIDYLINOSITOL-SPECIFIC PHOSPHOLIPASE C1* (*AtPLC1*) [12] and generates compounds such as inositol 1,4,5-trisphosphate (Ins(1,4,5)P3) [13] to transmit signals. In *Arabidopsis thaliana*, the MAP kinases AtMAPK4 and AtMAPK6 are activated in detached leaves in response to dehydration [14], and AtMAPK3, AtMAPK4, and AtMAPK6 are activated by hypo-osmolarity in cell suspension cultures [15,16]. 

The GSK3 family is an important protein kinase family involved in plant responses to osmotic stress, but the regulatory mechanisms of GSK3 in osmotic stress remain largely unknown. In *Arabidopsis*, the GSK3 kinase BRASSINOSTEROID-INSENSITIVE 2/ARABIDOPSIS THALIANA SHAGGY-RELATED KINASE 21 (BIN2/AtSK21) activates SnRK2.2/SnRK2.3 by phosphorylation [17], whereas BIN2 is inhibited by the PP2Cs ABI1 and ABI2 through dephosphorylation [18]. The transcription of *AtSK13* and *AtSK31*, two members of the *Arabidopsis* GSK3 family, is induced by osmotic stress [19]. BIN2 phosphorylates and stabilizes transcription factor RD26 to promote drought stress response in *Arabidopsis* [20]. *TaSK5*, isolated from winter wheat, shows high similarity to *Arabidopsis* GSK3 family members *AtSK11*, *AtSK12*, and *AtSK13*, which mediated drought tolerance in transgenic *Arabidopsis* [21]. In rice, T-DNA tagged knockout mutants of *OsGSK1* show enhanced tolerance to drought stress [22]. These findings suggest that certain GSK3 family members may play roles in plant responses to osmotic stress.

Root tips are crucial regions for sensing water in the soil and regulating root growth accordingly [2,23,24,25]. To investigate the possible roles of GSK3s in plant responses to osmotic stress, we examined the expression patterns of 10 GSK3 family members, and observed that *AtSK11*, *AtSK12*, and *AtSK13* are highly expressed in root tips. Using different concentrations of PEG to mimic osmotic stress conditions, we determined that the root growth of wild-type (WT) and *atsk11atsk12* plants was enhanced under the mild stress condition (−0.4 MPa) compare to the mock condition (−0.25 MPa); the enhancement effect on root growth in *atsk11atsk12* seedlings was much stronger than that of the WT, indicating a negative role of *AtSK11* and *AtSK12* in the mild stress-induced root growth response. RNA-seq analysis revealed 10 differentially expressed genes’ (DEGs) response to −0.4 MPa treatment, which may function downstream of *AtSK11* and *AtSK12*. Finally, we showed that the basic helix-loop-helix (bHLH) transcription factor LRL2 may mediate the regulation by *AtSK11* and *AtSK12* of these mild stress-responsive genes, thus shedding light on plant responses to mild osmotic stress.

## 2. Results

### 2.1. AtSK11 and AtSK12 Play a Negative Role in the Mild Osmotic Stress-Induced Root Growth Response

**Figure 1:**
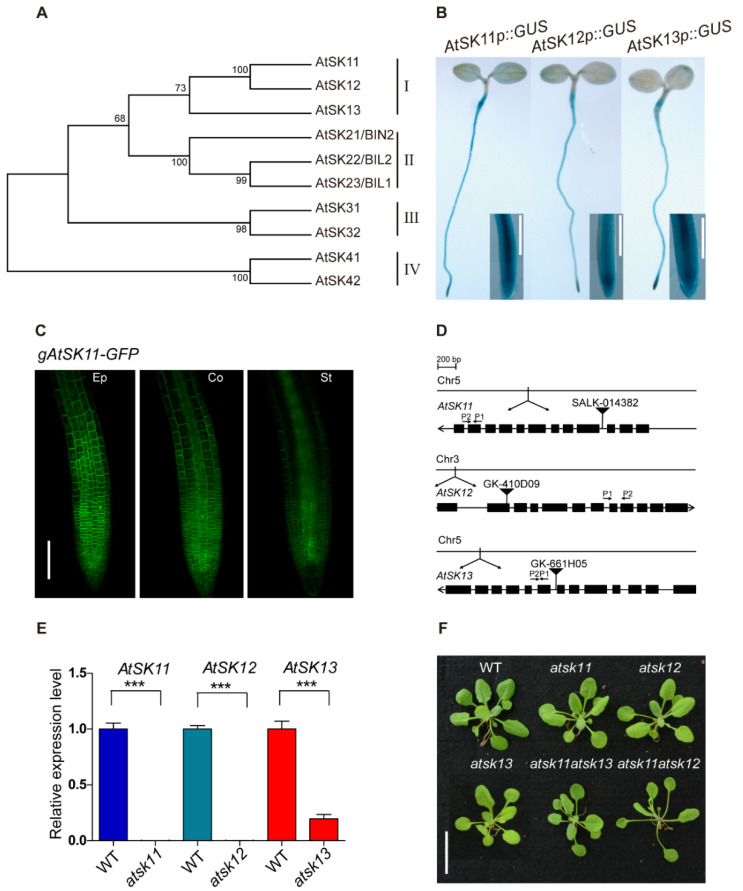
GLYCOGEN SYNTHASE KINASE 3 (GSK3) members AtSK11, AtSK12, and AtSK13 are highly expressed in the root tip. (**A**) Phylogenetic analysis of the *Arabidopsis* GSK3-like kinase gene family. Amino acid sequences were downloaded from the TAIR database, aligned with Clustal W. The evolutionary history was inferred using the maximum parsimony method. The percentages of replicate trees in which the associated taxa clustered together in the bootstrap test (1000 replicates) are shown. The evolutionary tree was constructed with MEGA7. (**B**) GUS staining of 5-day-old *AtSK11p::GUS*, *AtSK12p::GUS*, and *AtSK13p::GUS* seedlings. The rectangles show magnified views of the root tip images. Bar = 0.2 mm. (**C**) Subcellular localization of AtSK11-GFP in root tip cells. Images show epidermal (Ep), cortex (Co), and stele (St) cells in the root tips of 5-day-old *gAtSK11-GFP* transgenic seedlings. Bar = 0.1 mm. (**D**) Diagram of the T-DNA insertions in *atsk11*, *atsk12*, and *atsk13*. The qRT-PCR primer locations are labeled with arrows in the figures; P1 indicate forward primers and P2 indicate reverse primers. (**E**) Expression levels of *AtSK11*, *AtSK12*, and *AtSK13*, as detected by qRT-PCR, data were replicated in three times. Error bars indicate SD. Student’s *t*-test was used to determine the significance of difference between wild-type (WT) and mutant. Significant levels: *** *p* < 0.001. (**F**) Phenotypes of 3-week-old WT, *atsk11*, *atsk12*, *atsk13*, *atsk11atsk12*, and *atsk11atsk13* rosettes. Bar = 2 cm.

To explore the role of GSK3s in the response to osmotic stress in *Arabidopsis*, we performed promoter-GUS analysis to observe the expression patterns of the GSK3 family genes. The *Arabidopsis* GSK3 family contains 10 members, which belong to four clades (Figure 1A). GUS staining showed that only clade I members, namely *AtSK11*, *AtSK12*, and *AtSK13*, were highly expressed in root tips (Figure 1B, Figure S1A, Supplementary Materials). The root tip is an important region for sensing water [2,23,24,25]. Therefore, we focused on *AtSK11*, *AtSK12*, and *AtSK13* to investigate their function in osmotic stress responses. We first observed their subcellular localization in *AtSK11p::gAtSK11-GFP*, *AtSK12p::gAtSK12-GFP*, and *AtSK13p::gAtSK13-GFP* transgenic plants using confocal microscopy. GFP signals were detected in the cytosol of the epidermal, cortex, and stele cells in the root tips (Figure 1C, Figure S1B,C). Then, we obtained T-DNA knockout mutants and checked their effect on plant growth (Figure 1D,E). The *atsk11atsk12* and *atsk11atsk13* double mutants were further generated by crossing the corresponding single mutants. The obtained single mutants *atsk11*, *atsk12*, and *atsk13* and the double mutant *atsk11atsk13* did not show obviously different rosette phenotypes from the WT in the 3-week-old seedlings. Only *atsk11atsk12* had longer petioles and bolted earlier than the WT (Figure 1F). AtSK11 and AtSK12 function in perianth and gynoecium development. The double antisense mutant of *AtSK11* and *AtSK12* could not be obtained by crossing [26], and the *gsk3* sextuple mutation (*bin2-3bil1bil1*/*AtSK11-RNAi*, *AtSK12-RNAi*, *AtSK13-RNAi*) is seedling lethal [27], indicating that GSK3 clade I members are involved in plant fertility. We could not acquire the *atsk12atsk13* double mutant (we identified *atsk12*−/−, *atsk13*+/− seedlings through genotyping PCR, tested its 35 self-crossing descendants, and generated 25 *atsk12*−/−, *atsk13*+/− plants and 10 *atsk12*−/−, *atsk13*+/+ plants) or the *atsk11atsk12atsk13* triple mutant (we identified *atsk11*−/−, *atsk12*−/−, *atsk13*+/− seedlings through genotyping PCR, tested its 122 self-crossing descendants, and generated 84 *atsk11*−/−, *atsk12*−/−, *atsk13*+/− plants and 38 *atsk11*−/−, *atsk12*−/−, *atsk13*+/+ plants).

**Figure 2:**
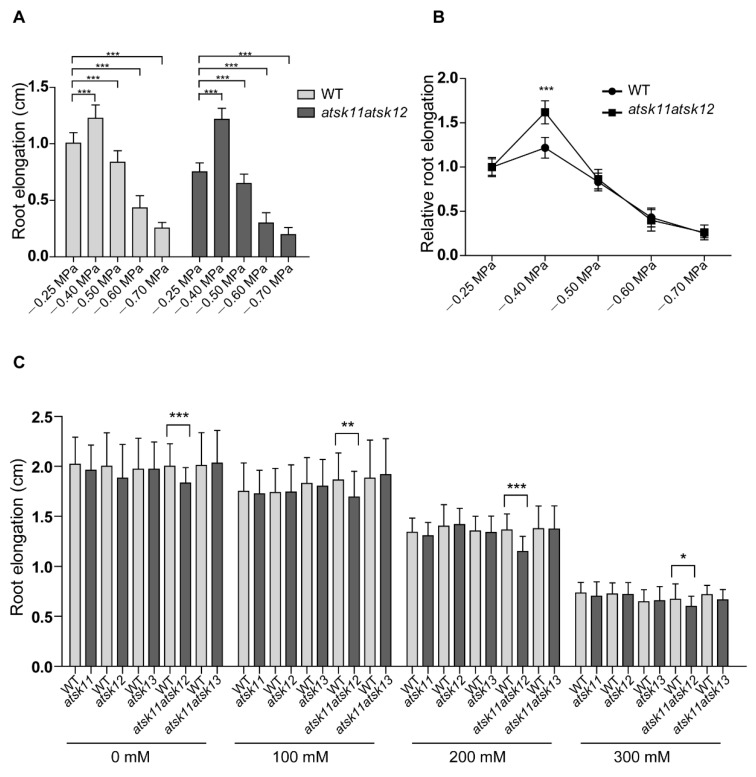
*AtSK11* and *AtSK12* inhibit the mild stress-induced root growth response. (**A**) Root elongation of WT and *atsk11atsk12* under polyethylene glycol (PEG) treatment. At least 150 seedlings were measured per genotype under each condition. Data are means of three independent biological replicates. Error bars indicate SD. Student’s *t*-test was used to determine the significance of difference between mock (−0.25 MPa) and other conditions, *** *p* < 0.001. (**B**) Relative root length of WT and *atsk11atsk12* under osmotic stress. The relative root length under −0.25 MPa was defined as “1”. Error bars indicate SD. Student’s *t*-test was used to determine the significance of difference between WT and mutant under each treatment condition. Significant levels: *** *p* < 0.001. (**C**) Root length of WT and various *AtSK11*, *AtSK12*, and *AtSK13* mutants under mannitol treatment. At least 50 seedlings were measured per genotype under each condition. Data are means of three independent biological replicates. Student’s *t*-test was used to determine the significance of difference between WT and mutant under each treatment condition. Significant levels: * *p* < 0.05, ** *p* < 0.01, *** *p* < 0.001.

To investigate whether *AtSK11*, *AtSK12*, and *AtSK13* are involved in osmotic response, we tested the responses of single mutants *atsk11*, *atsk12*, and *atsk13* and double mutants *atsk11atsk12* and *atsk11atsk13* to PEG and mannitol treatment. When we used PEG to mimic osmotic stress conditions, WT and *atsk11atsk12* showed stimulated root growth under the mild stress condition (−0.4 MPa) and reduced root growth under relatively strong osmotic stress conditions (−0.5, −0.6, and −0.7 MPa) compared to the mock condition without PEG (−0.25 MPa) (Figure 2A). The *atsk11atsk12* mutant was more sensitive in promoting root growth under the −0.4 MPa treatment than the WT (Figure 2B), which indicates a negative role for *AtSK11* and *AtSK12* in this mild stress response. In addition, the root length of the other mutants (*atsk11*, *atsk12*, *atsk13*, *atsk11atsk13*) and *gAtSK11-Flag* and *gAtSK12-Flag* transgenic lines did not significantly differ from the WT under mock and treatment conditions (Figure S2, Supplementary Materials). However, under mannitol treatment, the WT and these mutants showed reduced root growth compared to the mock condition, which is different from PEG-mimicked osmotic stresses (Figure 2C). These results indicate that *AtSK11* and *AtSK12* play a negative role in the mild stress (−0.4 MPa)-induced root growth regulation.

To investigate whether the protein levels of AtSK11 and AtSK12 are regulated by osmotic stress, we first performed immunoblot analysis using protein samples from the *gAtSK11-Flag* and *gAtSK12-Flag* roots. The results indicated that AtSK11 and AtSK12 protein levels were similar under different conditions in the same time point (Figure S3A–D, Supplementary Materials). Therefore, the tested osmotic stress conditions may not influence the protein levels of AtSK11 and AtSK12 in the indicated time point.

### 2.2. Identification of the Mild Osmotic Stress-Responsive Genes Regulated by AtSK11 and AtSK12

**Figure 3:**
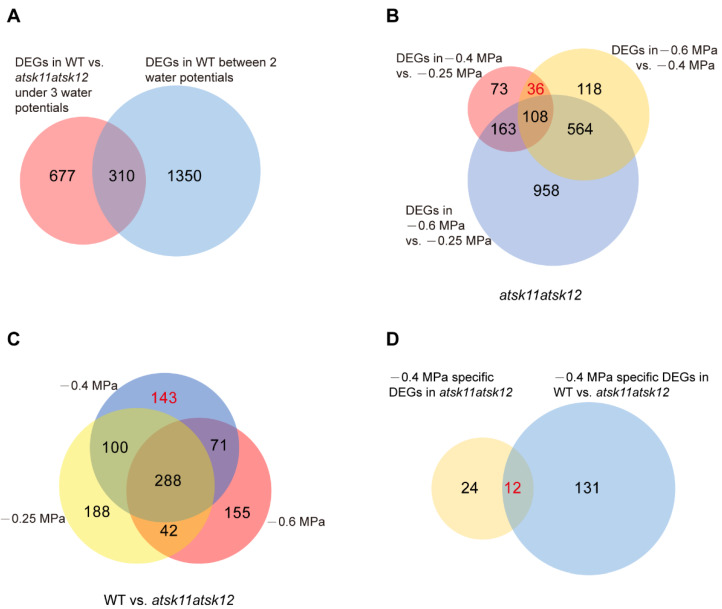
The mild osmotic stress-responsive genes regulated by *AtSK11* and *AtSK12* were identified through DEG analysis. (**A**) Venn diagram of two sets of DEGs, including all DEGs in WT vs. *atsk11atsk12* under three water potentials (−0.25 MPa, −0.4 MPa, and −0.6 MPa) and all DEGs in WT between two water potentials (−0.25 vs. −0.4 MPa, −0.4 MPa vs. −0.6 MPa, −0.25 MPa vs. −0.6 MPa). (**B**) Venn diagram of DEGs in *atsk11atsk12* between two water potentials. The number of −0.4 MPa-specific DEGs (36) in *atsk11atsk12* is indicated in red. (**C**) Venn diagram of DEGs of WT vs. *atsk11atsk12* under three water potentials. The number of −0.4 MPa-specific DEGs (143) in WT vs. *atsk11atsk12* is indicated in red. (**D**) Venn diagram of two sets of genes, including −0.4 MPa-specific DEGs (36) in *atsk11atsk12* and −0.4 MPa-specific DEGs (143) in WT vs. *atsk11atsk12*. The number of −0.4 MPa-responsive genes is indicated in red. The number of genes in each set is shown in the diagrams.

To identify downstream genes that are regulated by the mild (−0.4 MPa) osmotic stress through *AtSK11* and *AtSK12*, we performed RNA-seq of the *atsk11atsk12* and WT plants exposed to the mock condition (−0.25 MPa), a mild osmotic stress condition (−0.4 MPa), and a relatively strong osmotic stress condition (−0.6 MPa) using RNA samples from the 5-mm root tips. In total, we identified 1660 (310 + 1350) DEGs in WT between any two conditions (−0.25 vs. −0.4 MPa, −0.4 vs. −0.6 MPa, −0.25 vs. −0.6 MPa). These genes were considered stress-induced DEGs (Figure 3A). In addition, all DEGs between WT and *atsk11atsk12* under −0.25, −0.4, and −0.6 MPa comprised 987 (667 + 310) genes (Figure 3A). The overlapping genes of the two sets of DEGs (987 and 1660) contained 310 genes, which accounted for up to 18% of the stress-induced DEGs (1660) (Figure 3A), suggesting that *AtSK11* and *AtSK12* are highly involved in osmotic stress response. 

Next, we applied two approaches to identify the mild stress (−0.4 MPa)-responsive genes regulated by *AtSK11* and *AtSK12*. Because the root growth was induced only under −0.4 MPa (Figure 2A), the genes responsible for −0.4 MPa-induced root growth could be those differentially expressed in −0.4 MPa as compared to the mock condition (−0.25 MPa) and relatively strong osmotic stress (−0.6 MPa). In addition, because *atsk11atsk12* showed more sensitivity to −0.4 MPa in terms of induced root growth compared to Col-0 (Figure 2B), the mild osmotic stress-responsive genes regulated by *AtSK11* and *AtSK12* may be differentially expressed between *atsk11atsk12* and WT under −0.4 MPa treatment. Thus, first, by comparing the DEGs between any two water potential conditions in *atsk11atsk12* (Figure 3B), we identified 36 genes that were differentially expressed between −0.4 and −0.25 MPa and between −0.4 and −0.6 MPa, but not between −0.25 and −0.6 MPa, suggesting that these genes may be −0.4 MPa-specific DEGs in *atsk11atsk12*. Second, we compared three sets of DEGs including DEGs in WT vs. *atsk11atsk12* in −0.25 MPa, DEGs in WT vs. *atsk11atsk12* in −0.4 MPa, and DEGs in WT vs. *atsk11atsk12* in −0.6 MPa (Figure 3C). Among these, 143 DEGs were identified between WT and *atsk11atsk12* at −0.4 MPa, but not at −0.25 MPa or −0.6 MPa. Finally, we identified 12 genes overlapping between these 143 DEGs and the 36 DEGs (specifically induced by mild osmotic stress (−0.4 MPa) treatment in *atsk11atsk12*). These 12 genes were considered to be mild stress-responsive genes regulated by *AtSK11* and *AtSK12* (Figure 3D).

**Figure 4:**
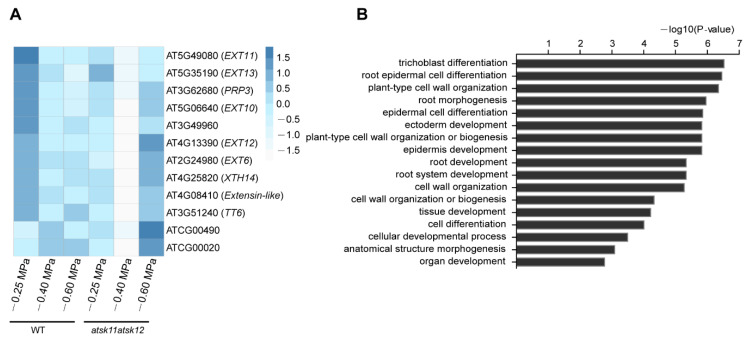
The expression of mild osmotic stress-responsive genes is altered in *atsk11atsk12*. The expression levels of mild osmotic stress (−0.4 MPa)-responsive genes were between *atsk11atsk12* and the WT under −0.4 MPa treatment. (**A**) Heatmap of −0.4 MPa-responsive genes. Values were normalized by Z-score. (**B**) GO enrichment analysis of −0.4 MPa-responsive genes.

The expression levels of these 12 mild stress-responsive genes were significantly downregulated in *atsk11atsk12* vs. WT under mild stress treatment (Figure 4A), indicating that they may work downstream of *AtSK11* and *AtSK12* to inhibit root growth under mild stress. Gene Ontology (GO) enrichment analysis suggested that these genes are involved in regulating root growth, including root hair differentiation, root epidermal cell differentiation, and plant cell wall organization (Figure 4B). Besides two genes in the chloroplast genome, the 10 remaining genes include five extensin genes (*EXT6*, *EXT10*, *EXT11*, *EXT12*, and *EXT13*), one extensin-like family member gene (*AT4G08410*), one proline-rich protein gene (*PRP3*), a xyloglucan endotransglucosylase/hydrolase protein gene (*XTH14*), a gene expressed in root hair cells (*AT3G49960*), and *TRANSPARENT TESTA 6* (*TT6*), encoding a flavanone 3-hydroxylase involved in flavonoid biosynthesis [28] (Figure 4A). We measured the expression levels of *EXT6*, *EXT10*, *EXT12*, and *EXT13* by qRT-PCR (Figure S4, Supplementary Materials); the results were in accordance with the RNA-seq data (Figure 4A). In summary, except for two chloroplast genome genes, we identified 10 mild stress-responsive genes regulated by *AtSK11* and *AtSK12* that might mediate changes in root growth under −0.4 MPa treatment.

### 2.3. Extensin Genes and TT6 Inhibit Root Growth in Response to Mild Osmotic Stress Treatment

**Figure 5:**
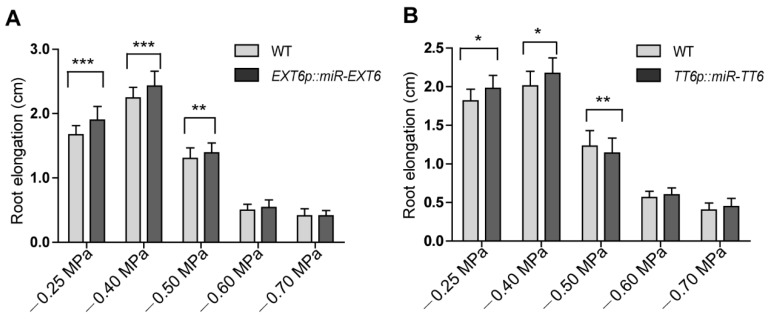
*EXTp::miR-EXT6* and *TT6p::miR-TT6* transgenic lines have longer roots under mild osmotic stress. Root lengths were compared between *EXTp::miR-EXT6* and *TT6p::miR-TT6* with the WT. (**A**) Root elongation in *EXT6p::miR-EXT6* transgenic plants under different water potentials. (**B**) Root elongation in *TT6p::miR-TT6* transgenic plants under different water potentials. At least 60 seedlings were measured per genotype. Data are means of three independent biological replicates. Error bars indicate SD. Student’s *t*-test was used to determine the significance of difference between WT and mutant under each treatment condition. Significant levels: * *p* < 0.05, ** *p* < 0.01, *** *p* < 0.001.

To investigate the roles of the mild stress-responsive genes in root growth, we created a set of knockdown lines of extensin genes (*EXT6*, *EXT10*, *EXT12*, and *EXT13*) and *TT6*, *PRP3*, *XTH14*, and *AT3G49960* by microRNA interference. Each artificial microRNA vector was driven by its own promoter. The gene expression level of each microRNA target in selected knockdown lines was reduced at least 50% (Figure S5A,B, Supplementary Materials). As these genes were downregulated by mild stress treatment in *atsk11atsk12* (Figure 4A), we predicted that the knockdown lines would have longer roots under the mild stress condition than the WT. We did observe that the transgenic lines *EXTp::miR-EXT6* (targeting *EXT6*, *EXT10*, *EXT12*, and *EXT13*) and *TT6p::miR-TT6* had longer roots under −0.25 and −0.4 MPa than the WT. By contrast, the root lengths in the knockdown lines of *PRP3*, *XTH14*, and *AT3G49960* were not significantly different from that of the WT at −0.4 MPa (Figure 5A,B, Figure S6A–C, Supplementary Materials). These findings suggest that the extensin genes and *TT6* may function downstream of *AtSK11* and *AtSK12* to inhibit the root elongation under mild osmotic stress (−0.4 MPa).

### 2.4. LRL2 Functions Downstream of *AtSK11* and *AtSK12* to Regulate −0.4 MPa-Responsive Gene Expression

**Figure 6:**
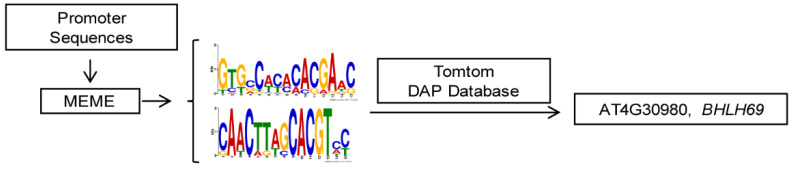
Diagram of the techniques used for promoter motif and transcription factor binding analyses.

**Figure 7:**
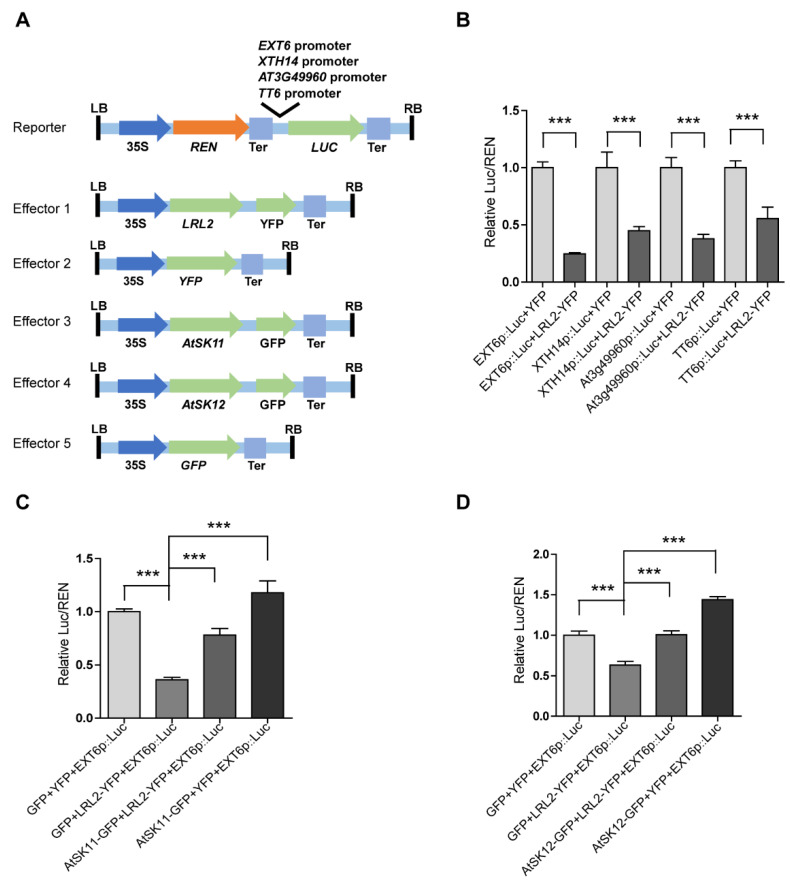
LRL2 inhibits the promoter activity of mild osmotic stress-responsive genes. (**A**) Diagrams of the dual-luciferase reporter and effector constructs. The *EXT6*, *XTH14*, *AT3G49960*, and *TT6* promoters were used to drive firefly *luciferase* (*Luc*). *35Sp::Renilla luciferase* (*REN*) was used as an internal control. For the effectors, the coding sequences of *LRL2*, *AtSK11*, and *AtSK12* were fused to *YFP* or *GFP* driven by the 35S promoter. (**B**) Relative Luc/REN ratios in *Nicotiana benthamiana* mesophyll cells co-transfected with different *Luc* reporters and *LRL2-YFP* or *YFP*. (**C**) Relative Luc/REN ratios in *N. benthamiana* mesophyll cells co-transfected with the *EXT6p::Luc* reporter and *LRL2-YFP*, *AtSK11-GFP*, or both constructs. (**D**) Relative Luc/REN ratios in *N. benthamiana* mesophyll cells co-transfected with the *EXT6p::Luc* reporter and *LRL2-YFP*, *AtSK12-GFP*, or both constructs. Data are means ± SD. Student’s *t*-test was used to determine the significance of difference. Significant levels: *** *p* < 0.001.

As kinases, AtSK11 and AtSK12 are unlikely to directly regulate gene expression; therefore, we reasoned that transcription factors must be involved to mediate their effects on the mild stress-responsive gene expression. To identify these transcription factors, we performed promoter motif analysis of the mild stress-responsive genes (Figure 6) and identified two conserved motifs. Motif 1 was enriched in the promoters of all 10 genes except *PRP3*; and motif 2 was enriched in the promoters of *EXT6*, *EXT10*, *EXT11*, *EXT12*, *EXT13*, *AT4G08410*, and *XTH14* (Tables S1 and S2, Supplementary Materials). We compared the two motifs to the motifs in the DAP-seq databases. The basic helix-loop-helix (bHLH) transcription factor bHLH69 was predicted to bind to motif 1 (with *p*-value of 2.13403 × 10^−5^ (Figure 6). bHLH69, also known as LJRHL1-LIKE 2/ DEFECTIVE REGION OF POLLEN 2 (LRL2/DROP2), regulates root hair and sperm cell development [29,30]. To examine whether LRL2 regulates the expression of the mild stress (−0.4 MPa)-responsive genes, we conducted dual-luciferase assays (Figure 7A) by transiently expressing various effector and reporter combinations in *Nicotiana benthamiana* mesophyll cells. We observed that LRL2 could inhibit the promoter activity of *EXT6*, *XTH14*, *AT3G49960*, and *TT6* (Figure 7B). Furthermore, when co-transfected with *AtSK11-GFP* or *AtSK12-GFP*, the inhibition of *EXT6* promoter activity by LRL2 was repressed (Figure 7C,D). These results suggested that LRL2 functions downstream of AtSK11 and AtSK12 to regulate the expression of mild stress-responsive genes in *Arabidopsis* roots.

## 3. Discussion

This study provides several lines of evidence to demonstrate that GSK3 family members *AtSK11* and *AtSK12* have a negative role in mediating the increased root growth in *Arabidopsis* induced by mild osmotic stress (−0.4 MPa) treatment. First, the promoter-GUS analysis of 10 GSK3 family members revealed that only *AtSK11*, *AtSK12*, and *AtSK13* are highly expressed in root tips, which are important for sensing water [2,23,24,25]. Second, using PEG to mimic osmotic stress conditions, the *atsk11atsk12* double mutant was more sensitive in promoting root growth to −0.4 MPa treatment than the WT, whereas root growth in the other mutants and overexpression lines was similar to that of the WT. Third, RNA-seq analysis identified 12 downstream genes that might be responsible for the mild stress-induced root growth in *atsk11atsk12*. Fourth, phenotyping of the knockdown mutants of the mild stress-responsive genes suggested that extensin genes and *TT6* might function downstream of *AtSK11* and *AtSK12* to inhibit root elongation under −0.4 MPa conditions. Fifth, promoter motif and transcription factor binding analyses of the 10 genes predicted that the bHLH transcription factor LRL2 may regulate the expression of stress-responsive genes. Indeed, LRL2 inhibits the promoter activity of *EXT6*, *TT6*, *XTH14*, and *AT3G49960*, as confirmed in a dual-luciferase assay, and AtSK11 and AtSK12 can largely suppress the inhibitory effect of LRL2 on the promoter activity of *EXT6* in a dual-luciferase assay.

It is likely that AtSK11 and AtSK12 function in the root growth response under mild stress conditions by regulating cell wall homeostasis. Among the 10 genes regulated by AtSK11/AtSK12 under mild stress conditions, five are extensin genes (*EXT6*, *EXT10*, *EXT11*, *EXT12*, and *EXT13*), and one is an extensin-like family gene (*AT4G08410*). Among the 20 classical extensins in *Arabidopsis*, nine are specifically expressed in roots based on the *Arabidopsis* eFP Browser (expression data for four extensins are not available in the database). Notably, four of the nine root-specific extensins are present in our list (Figure S8, Supplementary Materials) [31,32]. Extensin proteins are members of the cell wall hydroxyproline-rich glycoprotein (HRGP) superfamily that play key roles in cell wall homeostasis by functioning as scaffolds to facilitate self-assembly of the cell wall [33,34]. In addition to extensins, PRP3, a proline-rich structural cell wall protein [35], and XTH14, whose family members cleave xyloglucan chains and re-form bonds to the non-reducing ends of available xyloglucan molecules in primary cell walls, are also important for cell wall homeostasis [36]. It has been reported that the cell wall plays important roles in osmotic stress responses [3,34,37]. Osmotic stress leads to the accumulation of reactive oxygen species, resulting in cell wall stiffening through the crosslinking of cell wall glycoproteins such as extensins [3,38]. In addition, osmotic stress upregulates the expression of genes encoding xyloglucan-modifying enzymes to remodel the cell wall [3,38]. Therefore, it is likely that AtSK11 and AtSK12 take part in the root growth responses at −0.4 MPa by affecting cell wall homeostasis.

Several lines of evidence support the conclusion that LRL2 mediates the effect of AtSK11/AtSK12 on the expression of most mild stress-responsive genes. First, promoter motif and transcription factor binding analyses suggested that the transcription factor LRL2 binds to the promoters of all 10 mild stress-responsive genes except *PRP3*. Second, in dual-luciferase assays, LRL2 inhibited the promoter activity of *EXT6*, *TT6*, *XTH14*, and *AT3G49960*; this inhibitory function was repressed by co-transfection with *AtSK11* or *AtSK12* (Figure 7B–D). Third, LRL2 is expressed in roots and root hairs, and functions in root and root hair development [29]. GSK3 family members are also involved in root hair development [30], and *AtSK11* and *AtSK12* are highly expressed in roots, including root tips (Figure 1B,C, Figure S1B). Because AtSK11 and AtSK12 can inhibit LRL2 transcriptional activity in dual-luciferase assays, further studies could be conducted to explore how AtSK11 and AtSK12 regulate LRL2 activity.

In this study, we revealed the regulation mechanism of mild stress-induced root growth by GSK3-LRL2-cell wall-related genes, which could be useful in improving crop adaptation to drought stress. Osmotic stresses are caused by extreme water potentials. For sustainable agricultural production, it is critical to maintain soil water potential lower than −0.03 MPa but significantly greater than −1.5 MPa [39]. However, which water condition is best for plant growth remains elusive. Here, we demonstrated that the mild stress condition, −0.4 MPa, could facilitate root growth, and may thus be an ideal water potential for *Arabidopsis*, which could be useful information for future work to optimize agriculture irrigation.

## 4. Materials and Methods 

### 4.1. Plant Materials, Plant Growth, and Generation of Multiple Mutants 

*Arabidopsis thaliana* ecotype Col-0 was used as the wild type (WT) in this study. T-DNA insertion mutants *atsk11* (SALK_014382), *atsk12* (CS332559), and *atsk13* (CS340333) were obtained from the *Arabidopsis* Biological Resource Center (ABRC), Columbus, OH, USA. Homozygous mutants were identified by PCR, and the corresponding primers are listed in Table S5 (Supplementary Materials). 

Seeds were surface sterilized in 75% ethanol for 10 min, followed by absolute ethanol for 10 min. For general plant growth, the seeds were plated on 1/2 Murashige and Skoog (MS) medium (Phyto Technology Laboratories, Shawnee Mission, KS, USA) containing 0.5% sucrose and 0.8% agar. The pH of the medium was adjusted to 5.7 with KOH. After 2 days of stratification at 4 °C, the plates were transferred to a growth chamber (HiPoint, 740FLED, Gaoxiong, Taiwan, China) at 23 °C under a 16-h light/8-h dark cycle. After about 10 days of growth, the seedlings were moved to soil and grown in a greenhouse at 23 °C under a 16-h light/8-h dark cycle.

The *atsk11atsk12* and *atsk11atsk13* double mutants were generated by hybridization experiments with *atsk11* as female parent and *atsk12* and *atsk13* as male parents. Then, we identified the wild-type, heterozygous, and homozygous mutant plants (ratio about 1:2:1) by genotyping PCR. For the *atsk12atsk13* mutant, hybridization experiments were conducted with *atsk12* as female parent and *atsk13* as male parent. For the *atsk11atsk12atsk13* mutant, hybridization experiments were conducted with *atsk11atsk12* as female parent and *atsk13* as male parent. However, *atsk12atsk13* and *atsk11atsk12atsk13* were not identified. 

### 4.2. Osmotic Stress Treatment

For mannitol (Sigma-Aldrich, Saint Louis, MO, USA) treatment, seeds were plated on 1/2 MS medium, pH 5.7, containing 1% agar, vertical cultured for 4 days, and transferred to 1/2 MS medium containing 0, 100, 200, and 300 mM mannitol; the position of root tips was marked. Root length was measured after 3 days of growth. For PEG treatment, PEG (PEG8000, Sigma-Aldrich, Saint Louis, MO, USA)-infiltrated plates were prepared according to Verslues et al. [8] using 10 × 10 square Petri dishes and the PEG-infused plates were allowed to rest for 12 hours before use. The water potential of the plates was measured with a Dewpoint Potential Meter (WP4C, Pullman, WA, USA). Seeds were vertically grown in 1/2 MS medium (pH 5.7) containing 6 mM MES (Sigma-Aldrich, Saint Louis, MO, USA) and 1.5% agar (Sigma-Aldrich, Saint Louis, MO, USA) for 4 days, transferred to PEG-infiltrated plates; the position of root tips was marked and grown for 3 days. The plates were photographed, and root length from the marked position to the new position of the root tip was measured with ImageJ 1.48v software (National Institutes of Health, USA).

### 4.3. RNA-Seq Sample Preparation and Data Analysis 

Four-day-old WT and *atsk11atsk12* seedlings were transferred to PEG-infiltrated plates (−0.25 MPa, −0.4 MPa, −0.6 MPa). Following 3 days of growth, root tips (~5 mm) were collected from the plants. Each sequencing sample had two replicates. RNA was extracted from the samples using the TRIzol (Invitrogen, Dún Laoghaire, Dublin, Ireland) method. RNA-seq was performed using the Illumina HiSeq4000 platform (San Diego, CA, USA). Each sample generated approximately 23,000,000 clean reads (150 bp, PE). An average of 94.6% clean reads were mapped to the *Arabidopsis thaliana* genome from TAIR10 (ftp://ftp.arabidopsis.org/home/tair/Sequences/whole_chromosomes/) using TopHat2 [40] with default parameters. Differential expression analysis was performed with Cuffdiff software (Cambridge, MA, USA) (from the Cufflinks package [41], FDR < 0.05). The Fragments Per Kilobase Million (FPKM) value of WT and *atsk11atsk12* under three water potentials are listed in Tables S3 and S4 (Supplementary Materials). Heatmaps were generated with pheatmap in R, which normalizes FPKM values by Z-score. GO enrichment analysis was performed with agriGO (http://systemsbiology.cau.edu.cn/agriGOv2/index.php). The RNAseq Raw data have been deposited in the National Center for Biotechnology Information (NCBI) database with Bio-Project ID PRJNA636228.

### 4.4. Quantitative Real Time-PCR

For Figure 1E, RNA was extracted from the root of 10-day-old vertical growth seedlings of WT and *atsk11*, *atsk12*, *atsk13* homozygous mutants. For Figure S4, 4-day-old WT and *atsk11atsk12* vertical growth seedlings were transferred to PEG-infiltrated plates (−0.25 MPa, −0.4 MPa, −0.6 MPa), following 3 days of growth; root tips (~5 mm) were collected from each genotype under different treatment conditions. For Figure S5, RNA was extracted from the root of 10-day-old vertical growth seedlings of microRNA homozygous lines (T3). For Figure S7 (Supplementary Materials), RNA was extracted from the root of 10-day-old vertical growth seedlings of *gAtSK11-Flag* and *gAtSK12-Flag* transgenic homozygous plants (T3). RNA was extracted by TRIzol method, and the first strand cDNA was synthesized using the Takara first-strand cDNA synthesis kit (2641A, Beijing, China). cDNA was mixed with the SYBR Green Master Mix (Vazyme, Q511-02, Nanjing, Jiangsu, China) for qRT-PCR. The qRT-PCR primers were designed with NCBI Primer-Blast (http://www.ncbi.nlm.nih.gov/tools/primer-blast/), and are listed in Table S5. Three biological replicates were generated for each sample, and qRT-PCR results were analyzed using the 2^−ΔΔCt^ method.

### 4.5. Plasmid Construction and Plant Transformation

The plasmids used to produce Flag-, GFP-, and GUS-tagged proteins were generated in the *pCAMBIA 2306*, *pCAMBIA 2302*, and *pCAMBIA 1391* backbones, respectively. The promoter region of each gene included the region 1.5 kb upstream of the start codon, unless the sequence was <1.5 kb (in which case the longest sequence before the neighboring gene sequence was utilized). The genomic and promoter sequences of each gene were amplified from Col-0 genome DNA and cloned into the plasmid. Artificial microRNA was designed using the http://wmd3.weigelworld.org website and cloned into *pCAMBIA1300* driven by its native promoter. The primer sequences used in this study are listed in Table S5. The constructs were transformed into *Agrobacterium tumefaciens* GV3101 cells, and transgenic plants were generated using the floral dip method [42]. The T0 seeds were screened by germinating on 1/2 MS solid medium with antibiotic selection. For each artificial microRNA transformation, at least six individual transgenic T3 lines were selected. Following qRT-PCR, the lines with the most downregulated genes were chosen for phenotypic analysis (Figure S5).

### 4.6. GUS Staining and Immunoblot Analysis

For Figure 1B and Figure S1A, 5-day-old vertical growth of T3 *GSK3pro::GUS* transgenic homozygous lines were used for GUS staining, and at least three transgenic lines were analyzed. Histochemical staining of roots harboring the GUS reporter was performed as described [43].

For immunoblot analysis, the roots of *gAtSK11-Flag* and *gAtSK12-Flag* transgenic plants were ground to a fine powder in liquid nitrogen and solubilized with 5% SDS. After incubation at 95 °C for 5 min, the extracts were centrifuged at 12,000 rpm for 10 min. The resultant supernatant with 1× protein loading was separated on a 10% SDS-PAGE gel, then transferred to a nitrocellulose membrane (GE Healthcare Life Sciences, Marlborough, MA, USA), and the protein was detected with the corresponding antibodies. β-actin was used as an internal control. The gAtSK11-Flag and gAtSK12-Flag protein were detected by anti-Flag antibody (Abmart, Berkeley Heights, NJ, USA).

### 4.7. Confocal Microscopy

For Figure 1C and Figure S1B,C, *gAtSK11-GFP*, *gAtSK12-GFP*, and *gAtSK13-GFP* seedlings were grown vertically in 1/2 MS for 5 days, and the roots were imaged under a Leica TCS SP8 Confocal Microscope (Buffalo Grove, IL, USA). GFP signals were observed in the epidermal, cortex, and stele cells of the root tip. For each transgenic material, three transgenic lines were observed, and for each line, at least five plants were observed.

### 4.8. Motif enrichment and Promoter Binding Analyses 

The promoter sequences of the −0.4 MPa-responsive genes from 1500 bp upstream of their ATGs were extracted from the TAIR database and used to predict enriched motifs with an E-value < 0.05 in MEME software (V4.11.4, Reno, NV, USA) [44]. The enriched motifs were compared with known motifs from the DAP-seq database [45] using TOMTOM software (V4.11.4, Reno, NV, USA) [46] with a *p*-value < 10^−4^ and a *q*-value < 0.05.

### 4.9. Dual-Luciferase Assay

The dual-luciferase assay was performed as described [43]. Agrobacterium strain GV3101 cells carrying the constructs were transformed into 4-week-old *Nicotiana benthamiana* leaves. Three days after infiltration, total proteins were extracted from lower leaf mesophyll cells and subjected to dual-luciferase analysis (E1910; Promega, Beijing, China), and three biological replicates were conducted.

### 4.10. Statistical Analysis

For Figure 1E and Figure 7B–D, Figures S3B,D and S4, student’s *t*-test was used to determine the significance level of the difference. For Figure 2 and Figure 5, Figures S2 and S6, the F test was used to determine the variance and the two-tailed *t*-test with equal variance or unequal variance was used to determine the significance level of the difference. Asterisks indicate a significant difference (* *p* < 0.05, ** *p* < 0.01, *** *p* < 0.001).

## Supplementary Materials

Supplementary materials can be found at https://www.mdpi.com/1422-0067/21/11/3991/s1.

## Abbreviations

GO	Gene Ontology
PEG	Polyethylene glycol
ABA	Abscisic acid
DEGs	Differentially expressed genes
WT	Wild-type
AtSK	ARABIDOPSIS THALIANA SHAGGY-RELATED KINASE
GSK3	GLYCOGEN SYNTHASE KINASE 3
OSCA1	REDUCED HYPEROSMOLALITY-INDUCED CALCIUM INCREASE 1
PP2Cs	Protein serine/threonine phosphatase 2Cs
SnRK2s	SNF1-RELATED PROTEIN KINASE 2s
ABRE	ABA-responsive element
AREB/ABFs	ABRE-binding proteins/ABRE-binding factors
AtPLC1	PHOSPHATIDYLINOSITOL-SPECIFIC PHOSPHOLIPASE C1
Ins(1,4,5)P3	Inositol 1,4,5-trisphosphate
BIN2	BRASSINOSTEROID-INSENSITIVE 2
EXT	Extensin
PRP3	Proline-rich protein gene 3
XTH14	Xyloglucan endotransglucosylase/hydrolase 14
TT6	TRANSPARENT TESTA 6
LRL2/DROP2	LJRHL1-LIKE 2/ DEFECTIVE REGION OF POLLEN 2
bHLH	Basic helix-loop-helix
Luc	Firefly luciferase
REN	Renilla luciferase
HRGP	Hydroxyproline-rich glycoprotein
FPKM	Fragments Per Kilobase Million
