# Supplementary material for: AtSK11 and AtSK12 Mediate the Mild Osmotic Stress-Induced Root Growth Response in Arabidopsis
Source: Int J Mol Sci. 2020 Jun 2;21(11):3991. doi: 10.3390/ijms21113991 (PMC7312642; doi:10.3390/ijms21113991)
Supplement: Supplementary file 1 [file ijms-21-03991-s001.zip › Supplementary Figure legend.docx]

**Supplementary Figure legend**

**Supplementary Figure 1.** Expression patterns of GSK3 family members and information about the *AtSK11*, *AtSK12* and *AtSK13* T-DNA insertion mutants. (A) GUS staining of 5-day-old *AtSK21p::GUS*, *AtSK22p::GUS*, *AtSK23p::GUS*, *AtSK31p::GUS*, *AtSK32p::GUS*, *AtSK41p::GUS* and *AtSK42p::GUS* seedlings. Subcellular localization of AtSK12-GFP (B) and AtSK13-GFP (C) in root tip cells. Images show epidermis (Ep), cortex (Co), and stele (St) cells in the root tips of 5-day-old transgenic seedlings. Bar = 0.1 mm.

**Supplementary Figure 2.** Root elongation phenotypes of *AtSK11*, *AtSK12*, and *AtSK13* mutants and overexpression lines under osmotic stress. Root elongation of *atsk11atsk12* (A), *atsk11* (B), *atsk12* (C), *atsk13* (D), *atsk11atsk13* (E), *gATSK11-Flag* (F), and *gATSK12-Flag* (G) seedlings under osmotic stress. At least 120 seedlings were measured per genotype under each condition. Data are means of three independent biological replicates. Error bars indicate SD. Student’s t test was used to determine the significance of difference between WT and mutant under each treatment condition. Significant levels: ***P < 0.001.

**Supplementary Figure 3.** Protein levels of *AtSK11* and *AtSK12* in root of seedlings under –0.25 MPa, –0.4 MPa, and –0.6 MPa treatment. Protein levels of AtSK11-Flag (A) and AtSK12-Flag (C) in the root of 4-day-old transgenic seedlings following transfer to –0.25 MPa, –0.4 MPa, and –0.6 MPa plates at the indicated time points, as detected by immunoblot analysis. Anti β-actin was used as an internal control. AtSK11-Flag (B) and AtSK12-Flag (D) protein levels relative to β-actin were analyzed with ImageJ software. Error bars indicate SD. Student’s t test was used to determine the significance of difference between –0.25 MPa, –0.4 MPa, and –0.6 MPa in each time point, * P<0.05.

**Supplementary Figure 4.** Relative expression levels of *EXT6*, *EXT10*, *EXT12*, and *EXT13* under –0.25 MPa, –0.4 MPa, and –0.6 MPa treatment. Relative expression levels of *EXT6* (A), *EXT10* (B), *EXT12* (C), and *EXT13* (D) in WT and *atsk11atsk12* under –0.25 MPa, –0.4 MPa and –0.6 MPa treatment, as detected by qRT-PCR. Three replicates were conducted. Data are means ± SD. Student’s t test was used to determine the significance of gene expression difference between WT and *atsk11atsk12* under each treatment condition. Significant levels: **P < 0.01, ***P < 0.001.

**Supplementary Figure 5.** Expression levels of mild osmotic stress (–0.4 MPa)-responsive genes in artificial microRNAi lines. (A) Expression levels of *EXT6*, *EXT10*, *EXT12*, and *EXT13* in *EXT6p::miR-EXT6* in transgenic homozygote plants, as detected by qRT-PCR. (B) Expression levels of *TT6*, *PRP3*, *XTH14*, and *At3g49960* in artificial microRNAi lines, as detected by qRT-PCR. Three replicates were conducted. Data are means ± SD. Mock indicates plants transformed with empty vector.

**Supplementary Figure 6.** Phenotypic analysis of the roots of *XTH14p::miR-XTH14*, *PRP3p::miR-PRP3*, and *At3g49960p::miR-At3g49960* plants under osmotic stress. Root elongation of *XTH14p::miR-XTH14* (A), *PRP3p::miR-PRP3* (B), and *At3g49960p::miR-At3g49960* (C) plants under osmotic stress. At least 60 seedlings were measured per genotype under each condition. Data are means of three independent biological replicates. Error bars indicate SD. Student’s t test was used to determine the significance of difference between WT and mutant under each treatment condition. Significant levels: **P < 0.01, ***P < 0.001.

**Supplementary Figure 7.** Expression levels of *AtSK11* in *gAtSK11-Flag* transgenic plants and *AtSK12* in *gAtSK12-Flag* transgenic plants. Expression levels of *AtSK11* in *gAtSK11-Flag* transgenic plants and *AtSK12* in *gAtSK12-Flag* transgenic plants, as detected by qRT-PCR. Three replicates were conducted. Data are means ± SD. Mock indicates transgenic plants transformed with empty vector.

**Supplementary Figure 8.** The expression patterns of 16 classical extensin genes. The raw expression data were downloaded from the *Arabidopsis* eFP Browser, and the heatmap was generated using the pheatmap package in R. Expression data for At1g26250, At4g08400, At5g49080, and At3g28550 are not available in the database. The four –0.4 MPa-responsive extensin genes are shown in red.
