# Supplementary figures and images for: AtSK11 and AtSK12 Mediate the Mild Osmotic Stress-Induced Root Growth Response in Arabidopsis
Source: Int J Mol Sci. 2020 Jun 2;21(11):3991. doi: 10.3390/ijms21113991 (PMC7312642; doi:10.3390/ijms21113991)

**A**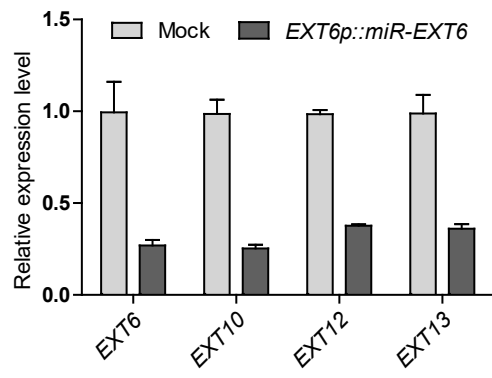**B**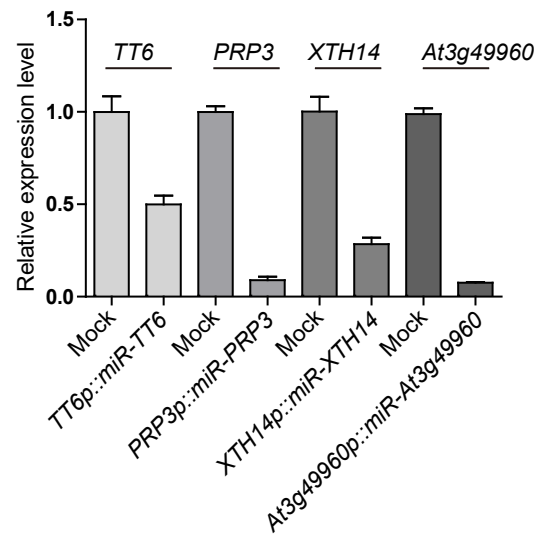

Supplement: Supplementary file 1 [file ijms-21-03991-s001.zip › Supplementary Figure 5.pdf]

**A**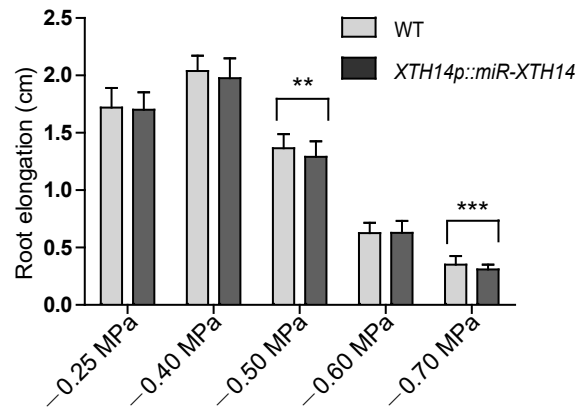**B**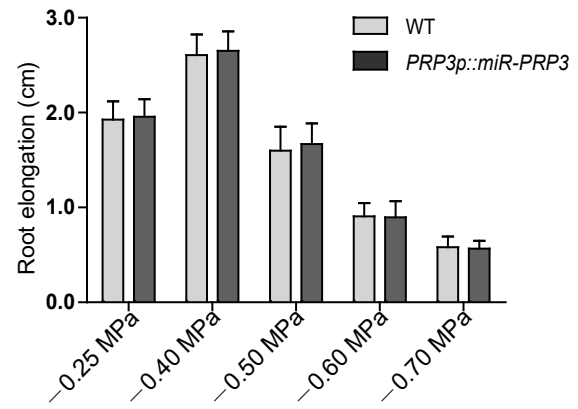**C**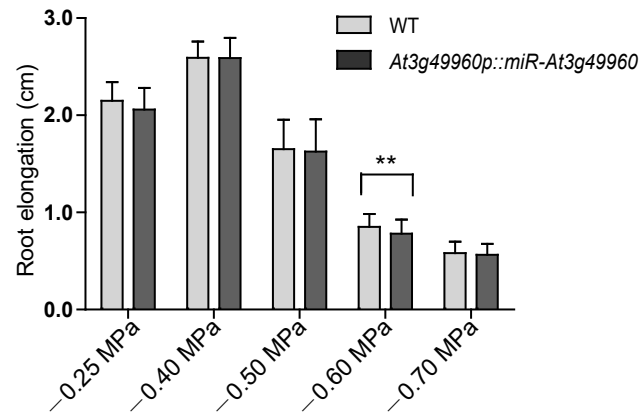

Supplement: Supplementary file 1 [file ijms-21-03991-s001.zip › Supplementary Figure 6.pdf]

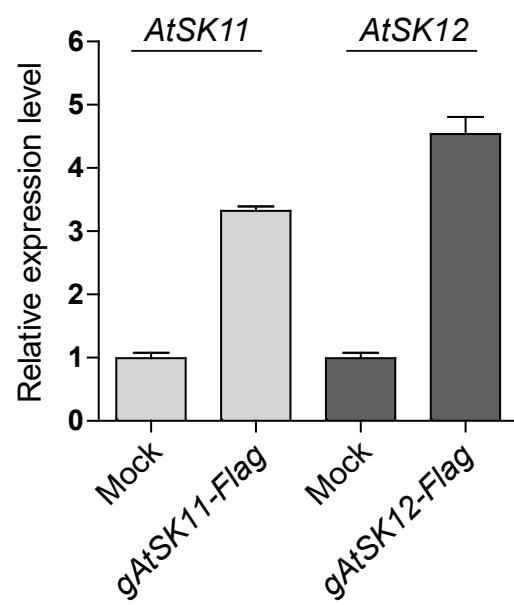

Supplement: Supplementary file 1 [file ijms-21-03991-s001.zip › Supplementary Figure 7.pdf]

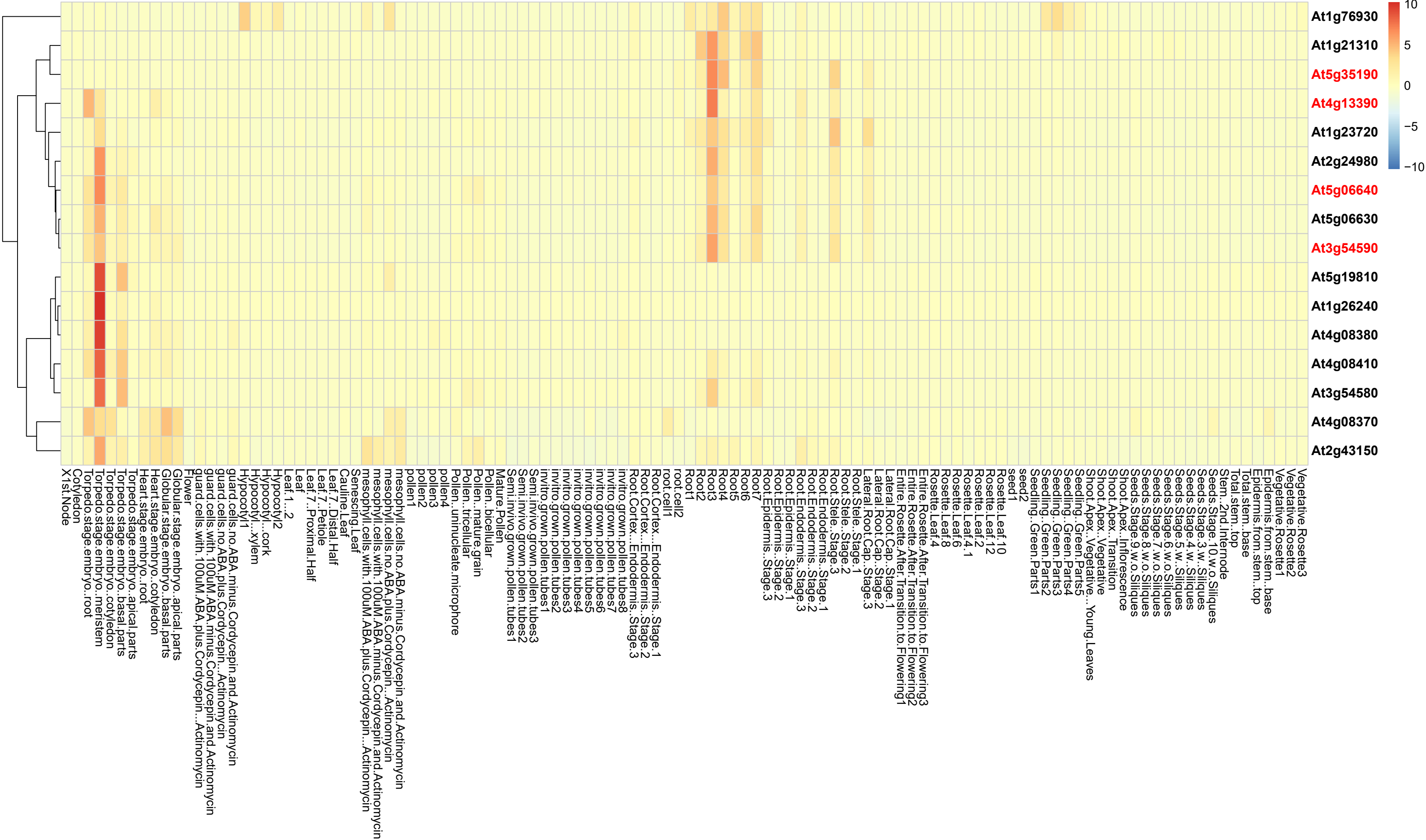

Supplement: Supplementary file 1 [file ijms-21-03991-s001.zip › Supplementary Figure 8.pdf]

**A**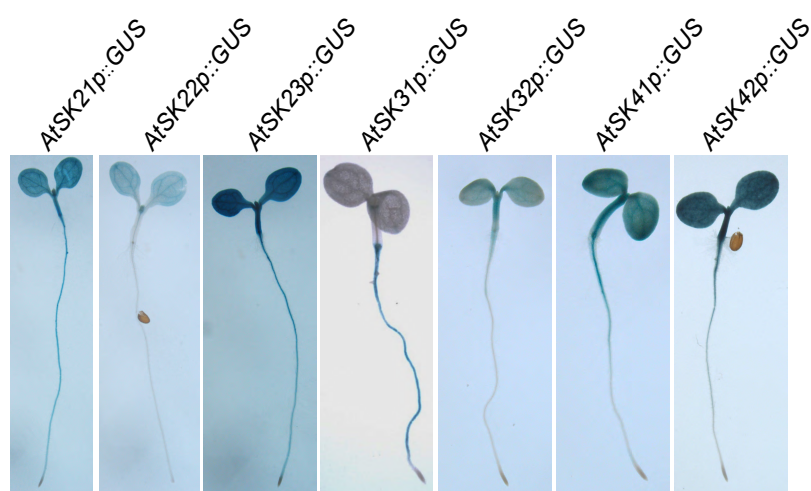**B***gAtSK12-GFP*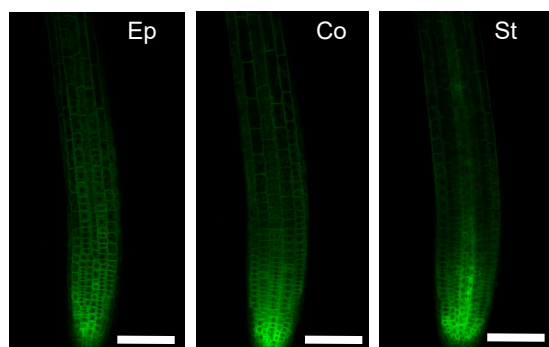**C***gAtSK13-GFP*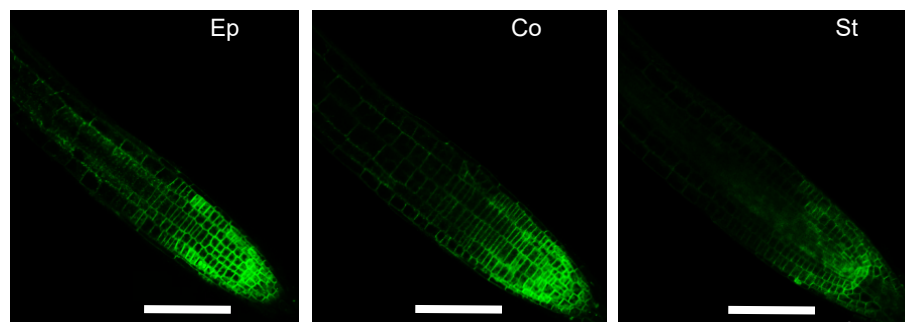

Supplement: Supplementary file 1 [file ijms-21-03991-s001.zip › Supplementary Figure 1.pdf]

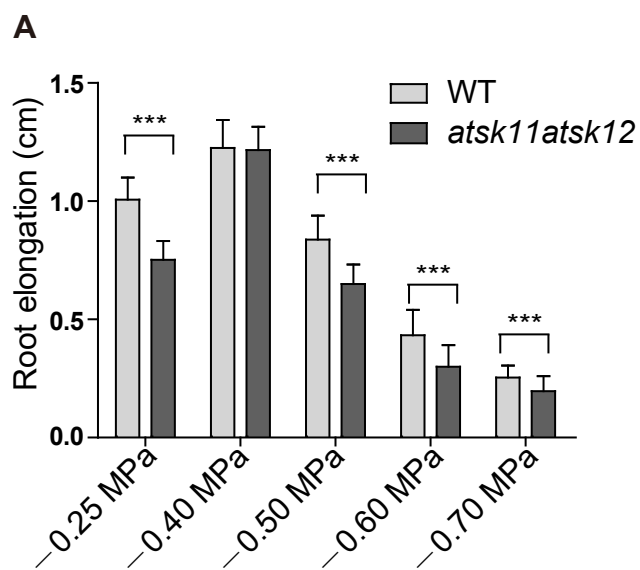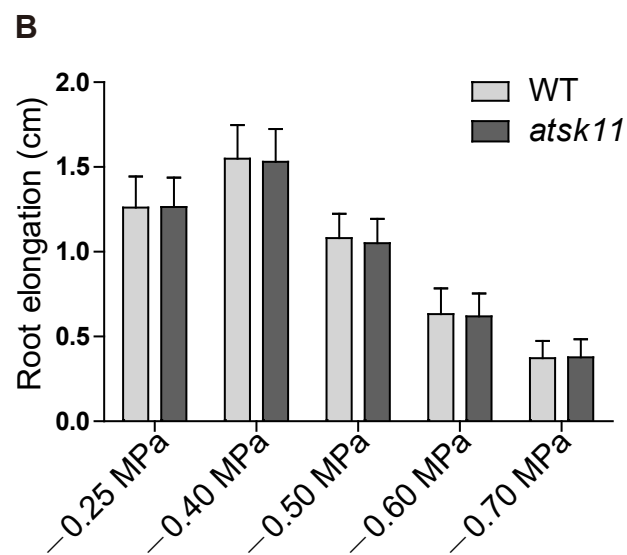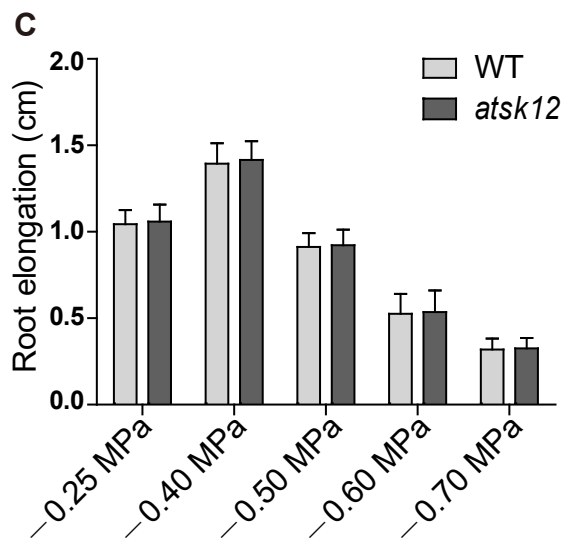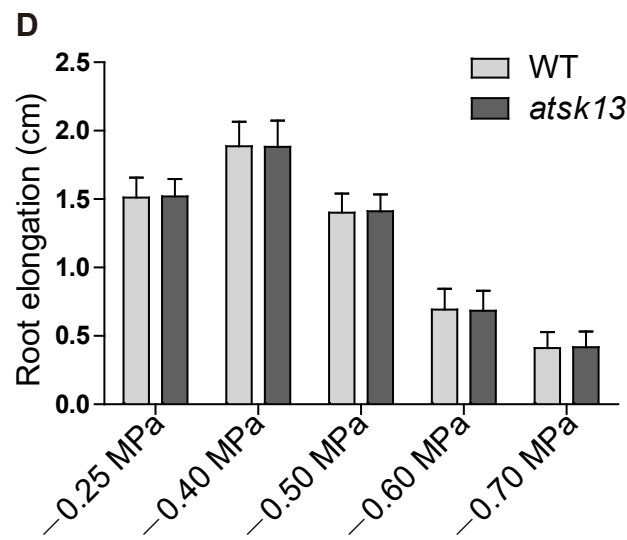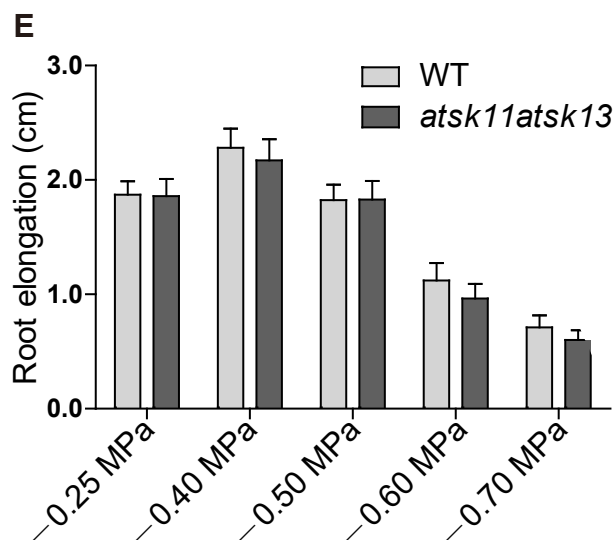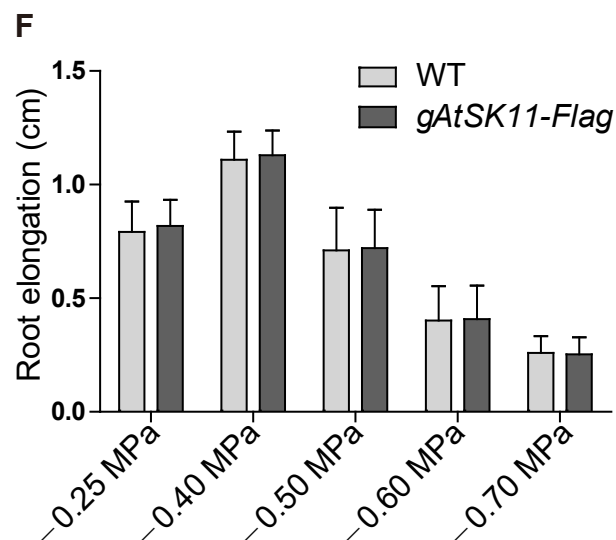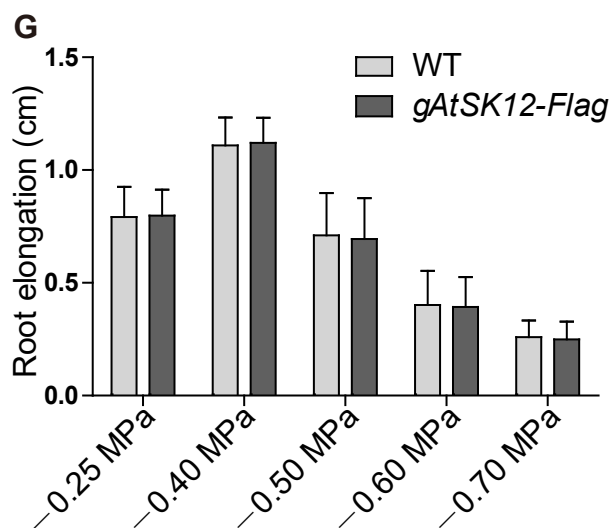

Supplement: Supplementary file 1 [file ijms-21-03991-s001.zip › Supplementary Figure 2.pdf]

**A**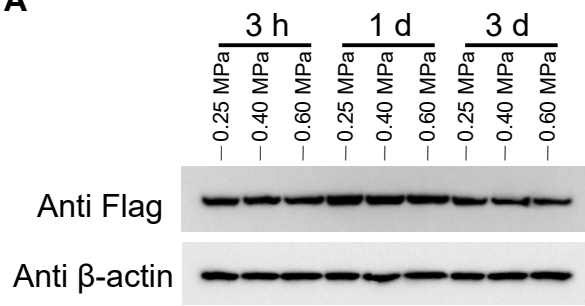**B**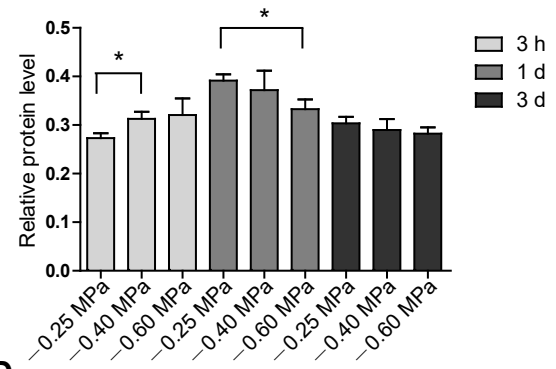**C**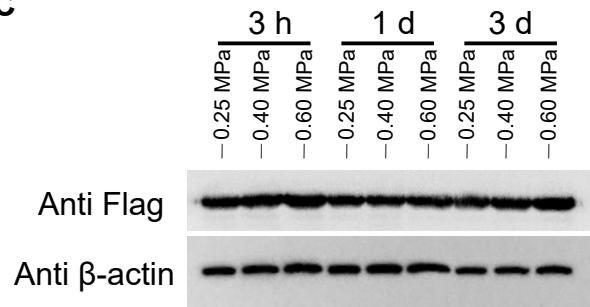**D**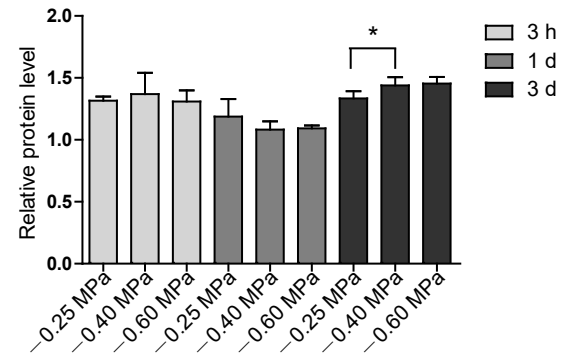

Supplement: Supplementary file 1 [file ijms-21-03991-s001.zip › Supplementary Figure 3.pdf]

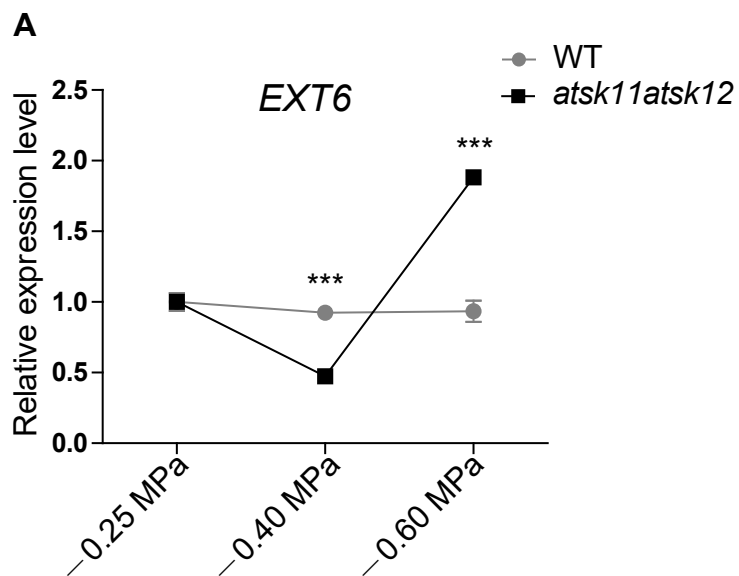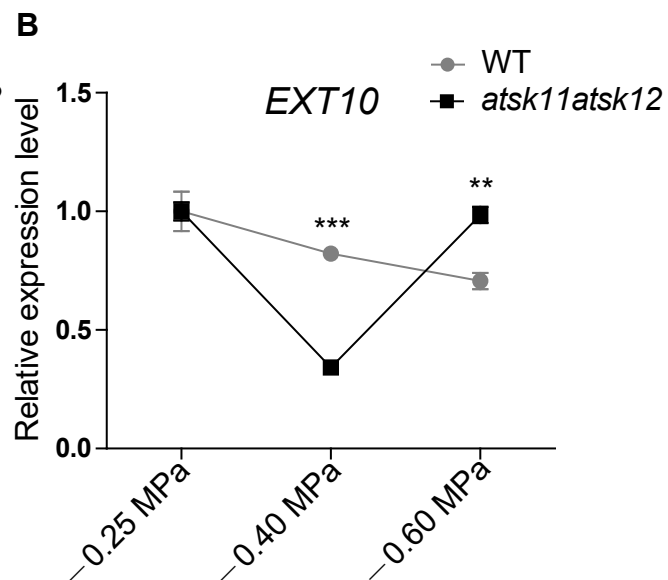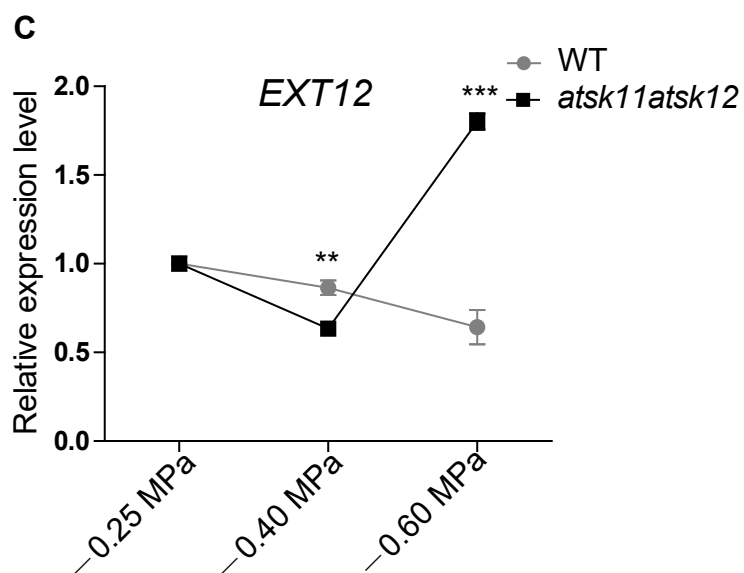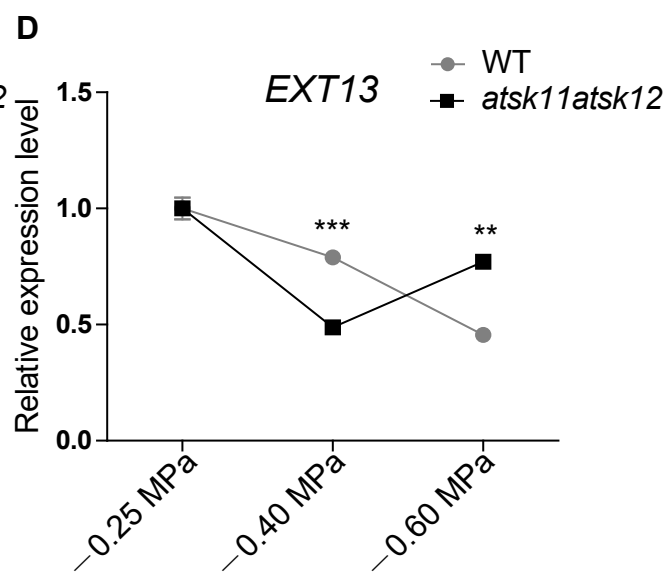

Supplement: Supplementary file 1 [file ijms-21-03991-s001.zip › Supplementary Figure 4.pdf]
